# Associations of Cardiometabolic Multimorbidity With All-Cause and Coronary Heart Disease Mortality Among Black Adults in the Jackson Heart Study

**DOI:** 10.1001/jamanetworkopen.2022.38361

**Published:** 2022-10-25

**Authors:** Joshua J. Joseph, Aakash Rajwani, Daniel Roper, Songzhu Zhao, David Kline, James Odei, Guy Brock, Justin B. Echouffo-Tcheugui, Rita R. Kalyani, Alain G. Bertoni, Valery S. Effoe, Mario Sims, Wen-Chi Wu, Gary S. Wand, Sherita H. Golden

**Affiliations:** 1 Department of Medicine, The Ohio State University Wexner Medical Center, Columbus; 2 Division of Public Health Sciences, Wake Forest School of Medicine, Winston-Salem, North Carolina; 3 Division of Biostatistics, College of Public Health, The Ohio State University, Columbus; 4 Division of Endocrinology, Diabetes and Metabolism, Johns Hopkins University School of Medicine, Baltimore, Massachusetts; 5 Department of Internal Medicine, Morehouse School of Medicine, Atlanta, Georgia; 6 Department of Medicine, University of Mississippi Medical Center, Jackson; 7 Department of Medicine, Warren Alpert Medical School of Brown University, Providence, Rhode Island

## Abstract

**Importance:**

A combination of diabetes, coronary heart disease (CHD), and stroke has multiplicative all-cause mortality risk compared with any individual morbidity in White populations, but there is a lack of studies in Black populations in the US.

**Objective:**

To examine the association of cardiometabolic multimorbidity (diabetes, stroke, and CHD) individually and collectively with all-cause and CHD mortality.

**Design, Setting, and Participants:**

This cohort study included Black adults in the Jackson Heart Study followed over a median of 15 years. Baseline examinations were performed between 2000 and 2004, with follow-up on all-cause and CHD mortality through May 31, 2018. Participants were categorized into mutually exclusive groups at baseline: (1) free of cardiometabolic morbidity, (2) diabetes, (3) CHD, (4) stroke, (5) diabetes and stroke, (6) CHD and stroke, (7) diabetes and CHD, and (8) diabetes, stroke, and CHD. Data were analyzed from 2019 to 2021.

**Exposure:**

Cardiometabolic disease alone or in combination.

**Main Outcomes and Measures:**

The main outcomes were all-cause mortality and CHD mortality. Cox models estimated hazard ratios (HRs) with 95% CIs adjusted for sociodemographic and cardiovascular risk factors.

**Results:**

Among 5064 participants (mean [SD] age, 55.4 [12.8] years; 3200 [63%] women) in the Jackson Heart Study, 897 (18%) had diabetes, 192 (4%) had CHD, and 104 (2%) had a history of stroke. Among participants with cardiometabolic morbidities, the crude all-cause mortality rates were lowest for diabetes alone (24.4 deaths per 1000 person-years) and highest for diabetes, CHD, and stroke combined (84.1 deaths per 1000 person-years). For people with only 1 cardiometabolic morbidity, risk for all-cause mortality was highest for people with stroke (HR, 1.74; 95% CI, 1.24-2.42), followed by CHD (HR, 1.59 (95% CI, 1.22-2.08) and diabetes (HR, 1.50; 95% CI, 1.22-1.85), compared with no cardiometabolic morbidities. There were also increased risks of mortality with combinations of diabetes and stroke (HR, 1.71; 95% CI, 1.09-2.68), CHD and stroke (HR, 2.23; 95% CI, 1.35-3.69), and diabetes and CHD (HR, 2.28; 95% CI, 1.65-3.15). The combination of diabetes, stroke, and CHD was associated with the highest all-cause mortality (HR, 3.68; 95% CI, 1.96-6.93). Findings were similar for CHD mortality, but with a larger magnitude of association (eg, diabetes, stroke, and CHD: HR, 13.52; 95% CI, 3.38-54.12).

**Conclusions and Relevance:**

In this cohort study, an increasing number of cardiometabolic multimorbidities was associated with a multiplicative increase in risk of all-cause mortality among Black adults, with a greater magnitude of association for CHD mortality.

## Key Points

**Question:**

Is there an association of increasing number of cardiometabolic comorbidities (diabetes, stroke, and coronary heart disease [CHD]) with all-cause mortality and CHD mortality in a population of US Black adults?

**Findings:**

In this cohort study of 5064 Black adults followed-up for 15 years, participants with cardiometabolic morbidities individually and collectively showed statistically significant multiplicative increased risks of all-cause and CHD mortality.

**Meaning:**

These findings suggest that a higher number of cardiometabolic comorbidities was associated with multiplicative increased risks of all-cause mortality and CHD mortality; thus, primordial and primary prevention of cardiometabolic morbidities are critical to reduce disparities in mortality.

## Teaser

This cohort study examines the association of cardiometabolic multimorbidity individually and collectively with all-cause and coronary heart disease mortality in Black adults in the Jackson Heart Study.

## Introduction

In the United States, inequities exist in life expectancy across racial and ethnic groups, with Black populations noted to have the shortest life expectancy at birth.^1^ Diabetes, stroke, and coronary heart disease (CHD) are 3 of the top 7 contributors to the differential life expectancy between non-Hispanic Black (hereafter, *Black*) and non-Hispanic White (hereafter, *White*) populations.^2^ Structural inequities lead to a higher prevalence of many cardiometabolic diseases in Black populations.^3,4,5^ For instance, the total diabetes prevalence is 17.4% in Black populations, compared with 13.6% in White populations.^6^ Although age-adjusted stroke death rates declined by 7% or more among all racial and ethnic groups between 2008 and 2018, rates remained higher in Black populations (52.3 deaths per 100 000 population; change since 2008, −12.7%) compared with White populations (35.9 deaths per 100 000 population; change since 2008, −11.4%).^7^ While Black and White populations have similar prevalence of CHD,^5,8,9^ Black populations have higher rates of mortality from stroke, CHD, and diabetes compared with White populations.^8^ The all-cause mortality rates for a history of diabetes, stroke, or myocardial infarction are approximately 16 per 1000 person-years for each condition among White populations, while a combination of these conditions is associated with a multiplicative mortality risk (32.0 per 1000 person-years with diabetes and myocardial infarction, 32.5 per 1000 person-years with diabetes and stroke, 32.8 per 1000 person-years with stroke and myocardial infarction, and 59.5 per 1000 person-years with diabetes, stroke, and myocardial infarction).^10^ Given the known higher rates of mortality from CHD, stroke, and diabetes among Black populations compared with White populations,^8^ it is paramount to examine the association of a combination of these cardiometabolic conditions with mortality among Black populations. Thus, we examined the association of diabetes, stroke, and CHD singularly and in combination with all-cause and CHD mortality among Black adults in the Jackson Heart Study (JHS). We hypothesized a multiplicative increase in mortality with greater combinations of morbidities.

## Methods

This cohort study was approved by the institutional review boards of all participating institutions, and written informed consent was obtained from all participants. This report followed the Strengthening the Reporting of Observational Studies in Epidemiology (STROBE) reporting guideline.

### Study Participants

The JHS is a prospective cohort study of 5306 Black adults (self-classified), aged 21 to 94 years from the tricounty area of metropolitan Jackson, Mississippi. Baseline examinations were performed between 2000 and 2004, with 2 subsequent follow-up examinations conducted in 2005 to 2008 and 2009 to 2013. The design of the study has been described elsewhere.^11^

In this prospective secondary analysis to examine the association of a combination of these cardiometabolic conditions with mortality among Black adults, 242 participants were excluded for missing data on exposures, outcomes, or primary covariates (age, sex, education, occupation, smoking, physical activity, alcohol intake, and waist circumference). After these exclusions, 5064 participants were included in the primary analysis. Additionally, 502 participants were excluded in an additional model including systolic blood pressure, estimated glomerular filtration rate (eGFR), low-density lipoprotein (LDL) cholesterol, and hemoglobin A_1c_ (HbA_1c_), for a final sample of 4562 participants in secondary models (eFigure in the Supplement).

### Ascertainment of Baseline Diabetes, CHD, or Stroke

Diabetes was defined as HbA_1c_ of 6.5% or greater (to convert to proportion of total hemoglobin, multiply by 0.01), fasting blood glucose of 126 mg/dL or greater (to convert to millimoles per liter, multiply by 0.0555), using diabetes medications, or a self-reported physician diagnosis.^12^ Fasting glucose was measured on a Vitros 950 or 250, Ortho-Clinical Diagnostics analyzer using standard procedures that met the College of American Pathologists accreditation requirement.^13^ A high-performance liquid chromatography system (Tosoh Corporation) was used to measure HbA_1c_ concentrations. History of CHD was defined as evidence of a previous myocardial infarction by electrocardiogram (ECG) based on Minnesota Code criteria (codes 1.1 and 1.2 plus 4.1-4.2, or 5.1-5.2) or history of physician-diagnosed myocardial infarction, percutaneous coronary intervention, or coronary bypass surgery. The definition of stroke was based on the history of stroke (by personal history, stroke signs, and symptoms ascertained by standardized questionnaires), transient ischemic attack, or carotid endarterectomy and/or angioplasty. Details of these procedures have been described previously.^14^

### Outcomes

The 2 outcomes of interest were all-cause mortality and CHD mortality. Methods for ascertaining all-cause mortality in the JHS have been described previously.^14^ Briefly, deaths were ascertained through a combination of active and passive surveillance. Annual follow-up included interviews with participants and next of kin to ascertain health events, such as cardiac events, hospitalizations, or death, through questionnaires completed by physicians and medical examiners or coroners and reviewed by the medical record abstraction unit to generate diagnosis information. Mortality was also assessed through death certificates requested from the Mississippi State Department of Health and National Death Index searches. Diagnoses were reviewed and adjudicated by trained medical personnel. The full protocol for CHD mortality is included in the eMethods in the Supplement. The *International Classification of Diseases, Ninth Revision *(*ICD-9*) and *International Statistical Classification of Diseases and Related Health Problems, Tenth Revision *(*ICD-10*) codes used for CHD mortality are shown in eTable 1 in the Supplement.

### Covariates

The covariates included demographics (age, sex), occupation (management or professional vs not), level of education (≥bachelor’s degree vs <bachelor’s degree attained), smoking status (current smoking vs not smoking), alcohol use (in the past 12 months vs no alcohol), and current prescription medication use. Waist circumference was calculated using the mean of 2 measurements around the umbilicus. Physical activity was categorized according to the American Heart Association 2020 cardiovascular health guidelines as poor, intermediate, or ideal health, as described previously.^15,16^ Fasting serum LDL cholesterol level was assayed using standard techniques and calculated by the Friedewald equation.^13^ eGFR was derived using the Chronic Kidney Disease Epidemiology Collaboration equation.^17^ Serum aldosterone was measured by radioimmunoassay (Siemens) and the intra-assay coefficients of variation were 8.7% for low concentrations and 6.2% for high concentrations.^16,18^

### Statistical Analysis

Baseline characteristics of participants are presented by baseline cardiometabolic multimorbidity status using the χ^2^ test for categorical variables, analysis of variance for parametric continuous variables, and Kruskal-Wallis test for nonparametric continuous variables. Baseline characteristics are also shown by all-cause mortality (eTable 2 in the Supplement) and CHD mortality (eTable 3 in the Supplement) over follow-up using the χ^2^ test for categorical variables, 2-sample *t* test for normally distributed continuous variables, and Wilcoxon 2-sample nonparametric test for nonnormally distributed continuous variables. Time to all-cause mortality was defined based on the adjudicated date. We censored data for participants at the time of study participation drop out or the end of study follow-up (May 31, 2018). We created cardiometabolic multimorbidity groups by categorizing participants in 8 mutually exclusive groups (participants without a history of diabetes, CHD, or stroke [reference group]; only diabetes; only stroke; only CHD; diabetes and stroke; diabetes and CHD; stroke and CHD; and diabetes, stroke, and CHD). Cox proportional hazards models were used to examine the associations of cardiometabolic multimorbidity with death. Based on prior analyses, covariates were selected a priori,^10,16,18^ and multivariable modeling was performed with sequential adjustment as follows: model 1 adjusted for age, sex, education, current occupation status, smoking, physical activity, alcohol use, and waist circumference; model 2: model 1 plus systolic blood pressure, eGFR, LDL, and HbA_1c_.

Additional adjustments were performed for aldosterone and aldosterone plus use of angiotensin converting enzyme–inhibitor, angiotensin-receptor blocker, mineralocorticoid receptor antagonist, statins, and aspirin (eTable 4 and eTable 5 in the Supplement). The proportional hazards assumption was assessed using Schoenfeld residuals, and no significant violations were noted. Direct adjusted survival curves (adjusted for model 2) were plotted by cardiometabolic multimorbidity groups. Statistical significance was defined as 2-sided α < .05 in the main analysis. Analyses were performed using SAS statistical software version 9.4 (SAS Institute). Data were analyzed from 2019 to 2021.

## Results

Among 5064 participants (mean [SD] age, 55.4 [12.8] years; 3200 [63%] women) 897 (18%) had diabetes, 192 (4%) had CHD, and 104 (2%) had a history of stroke (Table 1). Individuals with vs without diabetes, stroke, and CHD were older, more likely to be men, and less educated and had a higher prevalence of cardiovascular risk factors (eg, higher waist circumference, systolic blood pressure, glucose, HbA_1c_, and hypertension prevalence) except for lower diastolic blood pressure (Table 1). Similar findings were shown for individuals who had all-cause mortality (eTable 2 in the Supplement) or CHD mortality (eTable 3 in the Supplement) over the course of follow-up. Additionally, participants with mortality over the follow-up periods had a higher prevalence of baseline diabetes, stroke, and CHD.

**Table 1.** Baseline Characteristics of Participants Classified by Baseline Cardiometabolic Multimorbidity

Characteristic	Participants, No. (%)									*P* value^a^
	Overall (N = 5064)	No diabetes, stroke, or CHD (n = 3629)	Diabetes (n = 897)	Stroke (n = 104)	CHD (n = 192)	Diabetes and stroke (n = 60)	CHD and stroke (n = 31)	Diabetes and CHD (n = 125)	Diabetes, stroke, and CHD (n = 26	
Age, mean (SD), y	55.4 (12.8)	53.1 (12.9)	59.6 (10.8)	63.6 (10.9)	61.1 (11.4)	63.7 (100)	65.1 (9.0)	64.9 (8.5)	66.4 (8.5)	<.001
Sex										
Men	1864 (37)	1338 (37)	288 (32)	43 (41)	92 (48)	24 (40)	13 (42)	53 (42)	13 (50)	.001
Women	3200 (63)	2291 (63)	609 (68)	61 (59)	100 (52)	36 (60)	18 (58)	72 (58)	13 (50)	
<High school education	1018 (20)	565 (16)	244 (27)	42 (40)	67 (35)	24 (40)	15 (48)	45 (36)	16 (62)	<.001
Occupation, working full time	1812 (36)	1378 (38)	287 (32)	25 (24)	56 (29)	20 (33)	9 (29)	34 (27)	3 (12)	<.001
Current smoking	665 (13)	470 (13)	94 (10)	22 (21)	44 (23)	6 (10)	7 (23)	19 (15)	3 (12)	<.001
AHA Poor Physical Activity^b^	2482 (49)	1649 (45)	511 (57)	64 (62)	106 (55)	32 (53)	22 (71)	79 (63)	19 (73)	<.001
Alcohol consumption	2327 (46)	1824 (50)	311 (35)	37 (36)	86 (45)	12 (20)	6 (19)	45 (36)	6 (23)	<.001
BMI, mean (SD)	31.8 (7.2)	31.1 (7.1)	34.2 (7.2)	30.2 (5.8)	30.6 (7.4)	34.4 (7)	31.0 (7.3)	33.9 (7.0)	32.4 (6.4)	<.001
Waist circumference, mean (SD), cm	100.8 (16.1)	98.5 (15.7)	108.6 (15.6)	100.1 (12.8)	98.9 (16)	109.6 (16.2)	102.4 (16)	107.7 (13.9)	106.2 (11.9)	<.001
Blood pressure, mean (SD), mm Hg										
Systolic	127.5 (16.8)	125.9 (16.3)	131 (16.6)	130.2 (18)	133.6 (19.8)	136.4 (18.8)	132.7 (17.7)	131.8 (17.5)	130.6 (18.5)	<.001
Diastolic	75.8 (8.8)	76.3 (8.6)	74.7 (8.5)	74.9 (9.6)	76.4 (9.9)	73 (8.8)	76.0 (11.0)	72.4 (9.7)	69.5 (10.3)	<.001
Hypertension^c^	2864 (57)	1707 (47)	709 (79)	85 (82)	143 (74)	56 (93)	28 (90)	114 (91)	22 (85)	<.001
LDL cholesterol, mean (SD), mg/dL, (n = 4495)	126.6 (36.5)	127.7 (36.3)	124.5 (37)	122.5 (37)	117.4 (37.5)	124.5 (37.4)	124.6 (30.2)	123.4 (36.4)	129.6 (36.6)	.008
eGFR, mean (SD), mL/min per 1.73 m^2^	85.9 (18.5)	87.3 (16.4)	84.7 (21.9)	78.3 (18.3)	80.9 (24)	76.8 (20.6)	74.0 (22.4)	77.7 (24)	70.1 (32.5)	<.001
Laboratory measures, median (IQR)										
Aldosterone, ng/dL	4.4 (2.6-7.2)	4.3 (2.5-6.9)	4.6 (2.9-8.1)	3.9 (2.5-7.2)	4.4 (2.3-7)	4.6 (2.2-9.4)	3.9 (2.4-9.0)	5.0 (3.3-7.9)	6.9 (4.1-10.0)	<.001
Fasting plasma glucose, mg/dL, (n = 4543)	91 (85-99)	89 (84-95)	122 (103-160)	92.5 (88-97)	91 (86-98)	124 (106-162)	95 (91-100)	124 (103-158)	189 (136-244)	<.001
HbA_1c_, %, (n = 4801)	5.7 (5.3-6.2)	5.5 (5.2-5.8)	7.2 (6.6-8.4)	5.7 (5.3-6.0)	5.6 (5.3-5.9)	6.9 (6.5-7.8)	5.8 (5.5-6.1)	7.3 (6.6-8.3)	7.9 (6.6-9.2)	<.001
Medication use										
ACEI	968 (19)	434 (12)	339 (38)	24 (23)	53 (28)	30 (50)	14 (45)	60 (48)	14 (54)	<.001
ARB	413 (8)	213 (6)	131 (15)	15 (14)	16 (8)	9 (15)	3 (10)	24 (19)	2 (8)	<.001
MRA	64 (1)	35 (1)	13 (1)	1 (1)	6 (3)	2 (3)	0 (0)	6 (5)	1 (4)	<.001
Statins	574 (11)	222 (6)	196 (22)	14 (13)	51 (27)	20 (33)	12 (39)	47 (38)	12 (46)	<.001

Abbreviations: ACEI, angiotensin converting enzyme inhibitor; AHA, American Heart Association; ARB, angiotensin receptor blocker; BMI, body mass index (calculated as weight in kilograms divided by height in meters squared); eGFR, estimated glomerular filtration rate; HbA_1c_, hemoglobin A_1c_; LDL, low-density lipoprotein; MRA, mineralocorticoid receptor antagonist.

SI conversion factors: To convert aldosterone to picomoles per liter, multiply by 27.74; cholesterol to millimoles per liter, multiply by 0.0259; glucose to millimoles per liter, multiply by 0.0555; HbA_1c_ to proportion of total hemoglobin, multiply by 0.01.

^a^ *P* values were calculated using χ^2^ for categorical variables, analysis of variance for parametric continuous variables, and Kruskal-Wallis test for nonparametric continuous variables.

^b^ Poor physical activity was defined by AHA 2020 guidelines. Physical activity was considered poor if participant achieved 0 minutes/week moderate intensity or vigorous intensity physical activity.

^c^ Hypertension was defined as systolic blood pressure of 140 mm Hg or greater, diastolic blood pressure of 90 mm Hg or greater, or use of antihypertensive therapy.

**Figure 1.**
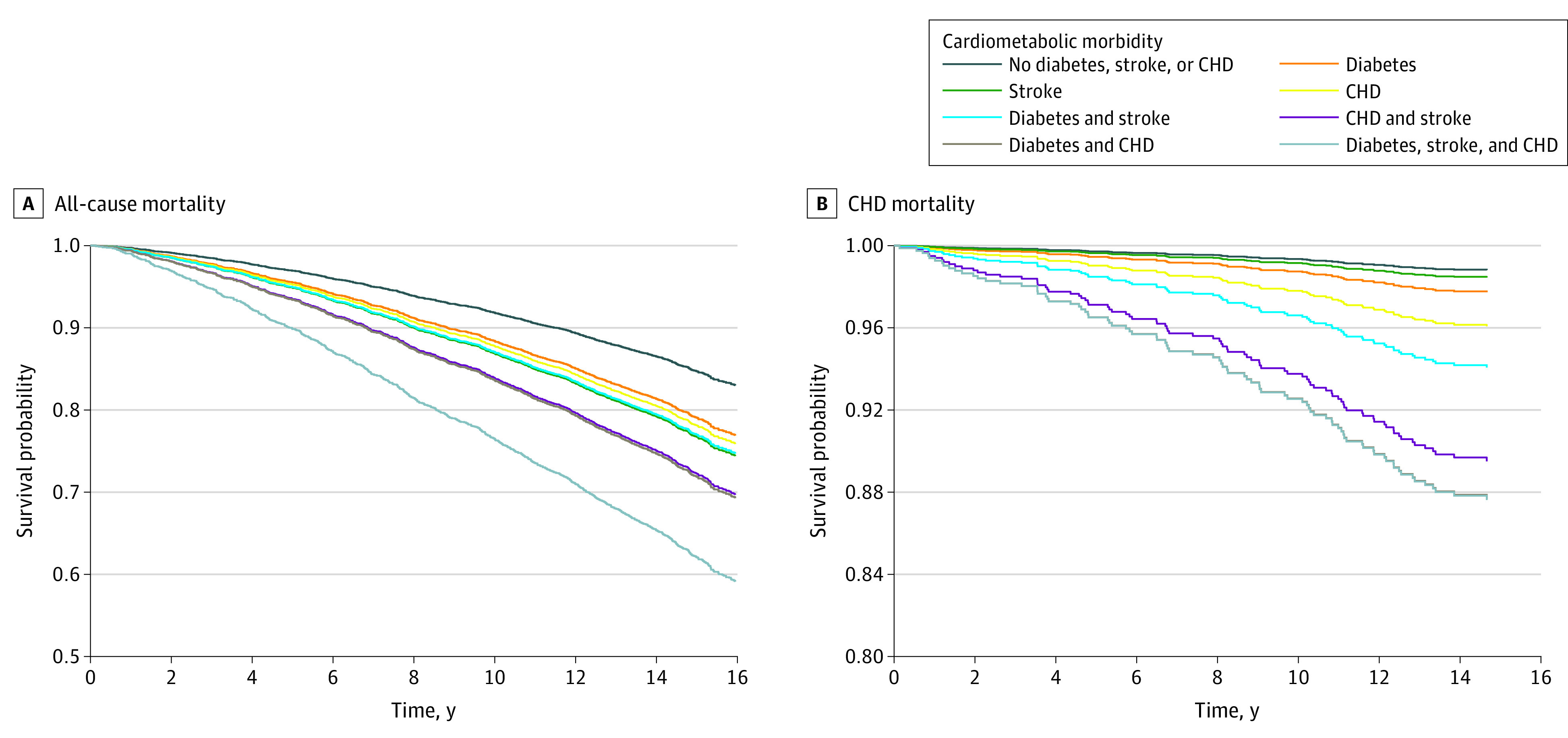
Adjusted Survival Curves for All-Cause and Coronary Heart Disease (CHD) Mortality by Cardiometabolic Multimorbidity in Black Adults The survival curves were adjusted for age, sex, education, occupation, smoking, physical activity, alcohol intake, waist circumference, systolic blood pressure, estimated glomerular filtration rate, low-density lipoprotein, and hemoglobin A_1c_.

**Figure 2.**
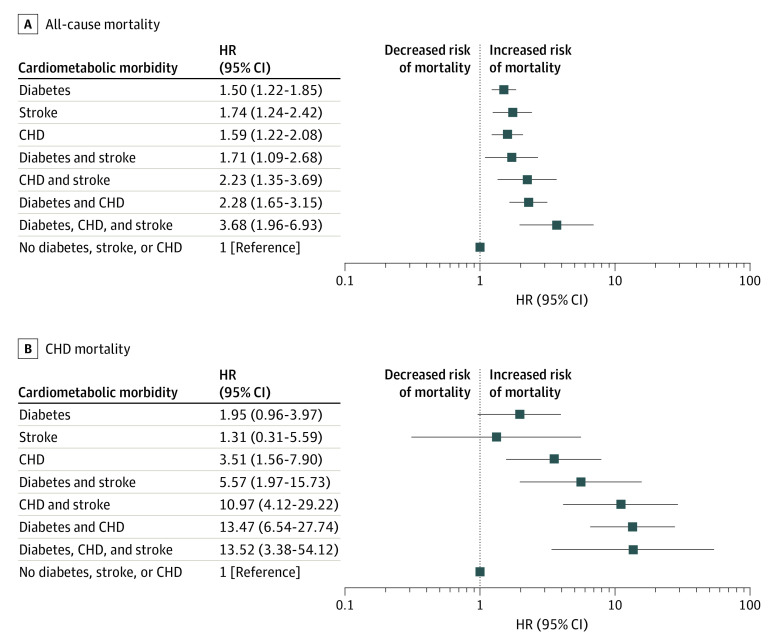
Forest Plots of the Association of Cardiometabolic Multimorbidity With All-Cause and Coronary Heart Disease (CHD) Mortality in Black Adults The models are adjusted for age, sex, education, occupation, smoking, physical activity, alcohol intake, waist circumference, systolic blood pressure, estimated glomerular filtration rate, low-density lipoprotein, and hemoglobin A_1c_. Analyses included 4562 participants due to missing data on low-density lipoprotein (4695 participants) and hemoglobin A_1c_ (4968 participants).

Over a median (IQR) follow-up period of 15.3 (14.3-16.1) years, there were 1068 all-cause deaths and 111 CHD deaths. Table 2, Figure 1A, and Figure 2A show the association of cardiometabolic multimorbidity with all-cause mortality. The crude mortality rate among participants without a history of diabetes, CHD, or stroke (reference group) was 9.65 (95% CI, 8.85-10.52) deaths per 1000 person-years. The crude mortality rates were 24.4 (95% CI, 21.78-27.34) deaths per 1000 person-years in participants with a history of diabetes, 32.95 (95% CI, 24.52-44.28) deaths per 1000 person-years in participants with a history of stroke, 29.07 (95% CI:23.11-36.56) deaths per 1000 person-years in participants with CHD, 40.18 (95% CI, 28.25-57.13) deaths per 1000 person-years in participants with diabetes and stroke, 48.02 (95% CI:29.85-77.25) deaths per 1000 person-years in participants with CHD and stroke, 48.62 (95% CI, 38.46-61.45) deaths per 1000 person-years in participants with diabetes and CHD, and 84.06 (95% CI, 54.80-128.92) deaths per 1000 person-years in participants with a history of diabetes, stroke, and CHD.

**Table 2.** Association of Cardiometabolic Multimorbidity With All-Cause Mortality Among Black Adults in the Jackson Heart Study

Outcome	Full cohort	No diabetes, stroke or CHD	Diabetes	Stroke	CHD	Diabetes and stroke	CHD and stroke	Diabetes and CHD	Diabetes, stroke, and CHD
Mortality No./total No.	1068/5064	515/3629	297/897	44/104	73/192	31/60	17/31	70/125	21/26
Crude incidence rate, No. per 1000 person-years (95% CI)	14.79 (13.93-15.70)	9.65 (8.85-0.52)	24.40 (21.78-27.34)	32.95 (24.52-44.28)	29.07 (23.11-36.56)	40.18 (28.25-57.13)	48.02 (29.85-77.25)	48.62 (38.46-61.45)	84.06 (54.80-128.92)
Model, HR (95% CI)^a^									
0	NA	1 [Reference]	2.58 (2.24-2.98)	3.53 (2.59-4.80)	3.1 (2.42-3.96)	4.32 (3.01-6.21)	5.08 (3.14-8.24)	5.37 (4.18-6.89)	10.14 (6.55-15.70)
1	NA	1 [Reference]	1.78 (1.54-2.07)	1.61 (1.17-2.20)	1.76 (1.38-2.26)	2.09 (1.45-3.02)	2.54 (1.56-4.14)	2.7 (2.10-3.47)	4.60 (2.96-7.15)
2	NA	1 [Reference]	1.50 (1.22-1.85)	1.74 (1.24-2.42)	1.59 (1.22-2.08)	1.71 (1.09-2.68)	2.23 (1.35-3.69)	2.28 (1.65-3.15)	3.68 (1.96-6.93)

Abbreviations: CHD, coronary heart disease; HR, hazard ratio; NA, not applicable.

^a^ Model 0 was unadjusted. Model 1 adjusted for age, sex, education, occupation, smoking, physical activity, alcohol intake, and waist circumference. Model 2 adjusted for model 1 plus systolic blood pressure, estimated glomerular filtration rate, low-density lipoprotein cholesterol, and hemoglobin A_1c_. Analysis included 4562 participants in model 2 due to missing data on low-density lipoprotein cholesterol level for 4695 participants and on hemoglobin A_1c_ for 4968 participants.

In the fully adjusted model, risk for all-cause mortality were significantly increased for participants with diabetes, (HR, 1.50; 95% CI, 1.22-1.85), stroke (HR, 1.74; 95% CI, 1.24-2.42), or CHD (HR, 1.59; 95% CI,1.22-2.08) alone. Risk was also increase in participants with diabetes and stroke (HR, 1.71; 95% CI, 1.09-2.68), CHD and stroke (HR, 2.23; 95% CI, 1.35-3.69), or diabetes and CHD (HR, 2.28; 95% CI, 1.65-3.15). The combination of diabetes, stroke, and CHD was associated with the greatest risk of all-cause mortality (HR, 3.68; 95% CI, 1.96-6.93).

Table 3, Figure 1B, and Figure 2B show the association of cardiometabolic multimorbidity with CHD mortality. In the fully adjusted model, there was no significant increased risk for CHD mortality for participants with diabetes (HR, 1.95; 95% CI, 0.96-3.97) or stroke (HR, 1.31; 95% CI, 0.31-5.59) alone, but there was increased risk for participants with CHD alone (HR, 3.51; 95% CI, 1.56-7.90). For participants with combined comorbidities, there was increased risk of CHD mortality for those with diabetes and stroke (HR, 5.57; 95% CI, 1.97-15.73), CHD and stroke (HR, 10.97; 95% CI, 4.12-29.22), diabetes and CHD (HR, 13.47; 95% CI, 6.54-27.74), or diabetes, stroke, and CHD (HR, 13.52; 95% CI, 3.38-54.12). Similar findings were noted after adjustment for aldosterone and use of medications affecting the renin-angiotensin-aldosterone system and statins (eTable 4 and eTable 5 in the Supplement).

**Table 3.** Association of Cardiometabolic Multimorbidity With CHD Mortality in the Jackson Heart Study

Participants	Full cohort	No diabetes, stroke or CHD	Diabetes	Stroke	CHD	Diabetes and stroke	CHD and stroke	Diabetes and CHD	Diabetes, stroke, CHD
Mortality, No./total No.	111/5064	30/3629	29/897	2/104	9/192	5/60	6/31	24/125	6/26
Crude incidence rate, No. per 1000 person-years (95% CI)	1.54 (1.28-1.85)	0.56 (0.39-0.80)	2.38 (1.66-3.43)	1.50 (0.37-5.99)	3.58 (1.86-6.89)	6.48 (2.70-15.57)	16.95 (7.61-37.73)	16.67 (11.17-24.87)	24.02 (10.79-53.46)
Model, HR (95% CI)^a^									
0	NA	1 [Reference]	4.24 (2.55-7.07)	2.65 (0.63-11.10)	6.39 (3.03-13.46)	11.50 (4.46-29.64)	30.4 (12.65-73.05)	29.77 (17.39-50.96)	42.85 (17.79-103.22)
1	NA	1 [Reference]	2.63 (1.56-4.44)	1.00 (0.23-4.27)	3.63 (1.71-7.69)	4.67 (1.79-12.14)	14.23 (5.86-34.56)	13.81 (7.98-23.91)	17.51 (7.17-42.74)
2	NA	1 [Reference]	1.95 (0.96-3.97)	1.31 (0.31-5.59)	3.51 (1.56-7.90)	5.57 (1.97-15.73)	10.97 (4.12-29.22)	13.47 (6.54-27.74)	13.52 (3.38-54.12)

Abbreviations: CHD, coronary heart disease; HR, hazard ratio; NA, not applicable.

^a^ Model 0 was unadjusted. Model 1 adjusted for age, sex, education, occupation, smoking, physical activity, alcohol intake, and waist circumference. Model 2 adjusted for model 1 plus systolic blood pressure, estimated glomerular filtration rate, low-density lipoprotein cholesterol, and hemoglobin A_1c_. Analysis included 4562 participants in model 2 due to missing data on low-density lipoprotein cholesterol for 4695 participants and on hemoglobin A_1c_ for 4968 participants.

## Discussion

In this prospective cohort study of Black adults, the risks of all-cause and CHD mortality were significantly increased with increasing numbers of cardiometabolic conditions (diabetes, stroke, and CHD), with the highest risk among participants with diabetes, stroke, and CHD. For participants with a combination of diabetes, stroke, and CHD, the unadjusted risk of all-cause mortality was increased approximately 10-fold and risk of CHD mortality was increased approximately 43-fold. With adjustment for established mortality risk factors, including systolic blood pressure, eGFR, LDL cholesterol, and HbA_1c_, risk of all-cause mortality was increased approximately 4-fold and risk of CHD mortality was increased approximately 14-fold. Thus, a combination of cardiometabolic conditions was associated with significantly increased risk of all-cause mortality and CHD mortality among Black adults, even after adjusting for established cardiovascular risk factors.

Comparing Black adults in the JHS to the predominantly White cohorts in the Emerging Risk Factors Collaboration (ERFC), the crude mortality rate in the referent group free of cardiometabolic morbidities was higher in the JHS, at 9.6 deaths per 1000 person-years, compared with 6.8 deaths per 1000 person-years in the ERFC, while crude mortality rates were higher for each cardiometabolic morbidity (24.4 deaths per 1000 person-years for diabetes, 32.9 deaths per 1000 person-years for stroke, and 29 deaths per 1000 person-years for CHD in the JHS vs approximately 16 deaths per 1000 person-years for each condition in the ERFC). In the JHS, crude mortality rates were 40.2 deaths per 1000 person-years for participants with diabetes and stroke, 48 deaths per 1000 person-years for participants with CHD and stroke, and 48.6 deaths per 1000 person-years for participants with diabetes and CHD, compared with approximately 32 deaths per 1000 person-years for each combination in ERFC. Participants with all 3 conditions in the JHS had a crude mortality rate of 84.1 deaths per 1000 person-years compared with approximately 60 deaths per 1000 person-years in the ERFC.^10^ In summary, the Black participants in the JHS had higher crude mortality rates per individual and combined cardiometabolic morbidity, but the multiplicative increase in risk was similar due to overall higher risk of mortality among Black participants free of cardiometabolic morbidities in the JHS. Both the JHS and ERFC included middle-aged participants (mean ages at baseline of 55 years in the JHS vs 52 years in the ERFC). The ERFC included more men than the JHS (57% vs 37%). The higher crude mortality rates among Black adults is concordant with higher rates of mortality among Black adults with CHD, stroke, or diabetes.^8^

### CHD, Diabetes, and Stroke Mortality

The prevalence of diabetes is increasing, with 14.7% of the US population with diagnosed or undiagnosed diabetes and higher rates in racial and ethnic minority groups, including Black populations.^19^ Individuals with diabetes are at higher risk of cardiovascular and all-cause mortality than those without diabetes.^20^ Racial disparities in mortality have been documented, with a 2-fold higher all-cause mortality in Black compared with White populations,^21^ although CVD and mortality rates are lower for Black individuals with equal access to care.^22^ For instance, among US veterans with diabetes, Black veterans had a 49% lower prevalence of CVD and a 13% lower incidence of mortality over 18 months than White veterans,^23^ which is consistent with data from other insured populations.^22,24^ This demonstrates the importance of glycemic and cardiovascular disease risk factor control, which are known to be less controlled among Black populations.^25,26^

CVD affects Black populations at an earlier age than White populations, and Black individuals with CHD have lower long-term survival than White individuals with CHD and higher rates of fatal CHD (2.2-fold higher for men; 1.6-fold higher for women), which may be mediated by a higher prevalence of risk factors.^27,28,29^ Since the mid-20th century, stroke mortality has decreased by 80%, but the racial disparity between Black and White adults remains, with 4- to 5-fold higher mortality among Black compared with White populations.^8^ The underlying cause of the higher stroke mortality is not completely known but may be attributable to higher stroke incidence and mortality after stroke events.^8^

### Interventions

The multiplicative increased risk of all-cause and CHD mortality seen in this US Black population is a call to action to prevent the development of cardiometabolic disease and advance the treatment of care of those with known cardiometabolic morbidities.^4^ Over 30 years since the Heckler report,^30^ and 20 years since the Unequal Treatment report,^31^ we are still grappling as a society and medical community with how best to address racial and ethnic disparities in chronic disease and life expectancy. Much of the discussion on prevention revolves around individual lifestyle change, but appreciating that individual lifestyle change occurs in the environment and context of a person’s living situation and is impacted by the social determinants of health (SDOH) is critical to addressing inequities.^32,33,34^ Thus, intervening upstream in the sociopolitical and economic context, including structural racism and discrimination, accessible quality education and health care, socioeconomic status, and healthier built environments, could help to improve midstream determinants like nonmedical health-related social needs (eg, social and community context, social risk, and lived personal experience), which, through impacting psychological and environmental stressors, as well as biological and psychological sequelae, would help to improve CVD prevention and outcomes.^33^ Recent evidence suggests that addressing the SDOH may reduce Black vs White disparities in CVD risk factors by as much as 50%.^35^ Thus, emerging interventions that address SDOH in addition to traditional CVD prevention are critical to cardiovascular equity.

One pillar of equity-based interventions is to address multiple levels of the socioecological model, including through community-engaged and community-based participatory research centered on equity to advance cardiometabolic health equity.^5,36^ More ideal levels of American Heart Association’s Life’s Simple 7 are associated with lower risk of diabetes, heart disease, and stroke.^37,38,39^ Unfortunately, community-based participatory research to improve all components of Life’s Simple 7 in US Black populations are lacking.^40^ The 24-week Black Impact Study^41^ focused on health education, physical activity, and addressing social needs to improve Life’s Simple 7 in Black men and showed a significant impact in improving cardiovascular health metrics. The FAITH! trial^42,43,44^ used an innovative app-based platform to advance Life’s Simple 7. Innovative solutions, such as expansion of equity-based efforts (eg, Black Impact and FAITH!), are urgently needed to advance cardiometabolic health equity. Policy approaches to eradicate structural inequities in historically marginalized communities would catalyze further gains toward eliminating health disparities,^3,4,5,32^ as evidenced by the Moving to Opportunity study,^45^ in which women using housing vouchers to move to a low-poverty census track had lower rates of class II and III obesity and HbA_1c_ levels more than 10 years later.^45^

Strengths of our study include a large, community-based, socioeconomically diverse, contemporaneous Black cohort with more than 15 years of follow-up to assess mortality. We used validated questionnaires to assess risk factors and a comprehensive documentation of diabetes over time, including fasting glucose, HbA_1c_, medication use, and self-reported physician diabetes diagnosis. We performed a comprehensive ascertainment of deaths.

### Limitations

This study has some potential limitations. First, the participants in the JHS are from 1 geographic area in the southeastern United States recruited from 4 recruitment pools (Atherosclerosis Risk in Communities participants, random selection, family members, and volunteers). Hence JHS participants may not be representative of all Black adults in the US, limiting generalizability. Second, the cardiometabolic morbidities were established at baseline with no adjustment for acquired morbidities over the course of the study. Third, baseline differences existed across the cardiometabolic multimorbidity groups, which we adjusted for in the models, but residual confounding may still exist. Fourth, statistical associations were interpreted without correction for multiple comparisons, as typical multiple comparison corrections assume tests are independent and are too conservative for correlated hypotheses, as have been performed. Fifth, while adjustments were made for socioeconomic status (ie, education and current occupation status) and lifestyle behaviors (ie, smoking and physical activity), we did not comprehensively examine potential mediators and confounders, including SDOH, psychosocial stressors, and lifestyle behaviors. Therefore, some caution is warranted in the interpretation of our study results.

## Conclusions

This cohort study reveals a novel association of an increasing number of cardiometabolic multimorbidities with a multiplicative increase in all-cause and CHD mortality risk among Black adults in the US, with a greater magnitude of association for CHD mortality. Given the increasing prevalence of obesity and type 2 diabetes in the US, along with recent increases in cardiovascular mortality, it is imperative to accelerate the development and implementation of preventive interventions to decrease cardiometabolic disease and advance treatment and care among those with cardiometabolic conditions.

## Notes

Supplement.
eMethods.
**eTable 1.** *ICD-9* and *ICD-10* codes for Jackson Heart Study Coronary Heart Disease Events **eTable 2.** Baseline Characteristics of Participants in the Jackson Heart Study by All-Cause Mortality **eTable 3.** Baseline Characteristics of Participants in the Jackson Heart Study by Coronary Heart Disease Mortality **eTable 4.** Association of Cardiometabolic Multimorbidity With All-Cause Mortality Among Black Adults in the Jackson Heart Study **eTable 5.** Association of Cardiometabolic Multimorbidity With Coronary Heart Disease Mortality Among Black Adults in the Jackson Heart Study **eFigure.** Exclusion Cascade
