# Supplementary material for: Associations of Cardiometabolic Multimorbidity With All-Cause and Coronary Heart Disease Mortality Among Black Adults in the Jackson Heart Study
Source: JAMA Netw Open. 2022 Oct 25;5(10):e2238361. doi: 10.1001/jamanetworkopen.2022.38361 (PMC9597394; doi:10.1001/jamanetworkopen.2022.38361)
Supplement: Supplement. — eMethods. eTable 1. ICD-9 and ICD-10 codes for Jackson Heart Study Coronary Heart Disease Events eTable 2. Baseline Characteristics of Participants in the Jackson Heart Study by All-Cause Mortality eTable 3. Baseline Characteristics of Participants in the Jackson Heart Study by Coronary Heart Disease Mortality eTable 4. Association of Cardiometabolic Multimorbidity With All-Cause Mortality Among Black Adults in the Jackson Heart Study eTable 5. Association of Cardiometabolic Multimorbidity With Coronary Heart Disease Mortality Among Black Adults in the Jackson Heart Study eFigure. Exclusion Cascade [file jamanetwopen-e2238361-s001.pdf]

Supplemental Online Content

Joseph JJ, Rajwani A, Roper D, et al. Associations of cardiometabolic multimorbidity with all-cause and coronary heart disease mortality among Black adults in the Jackson Heart Study. *JAMA Netw Open*. 2022;5(10):e2238361. doi:10.1001/jamanetworkopen.2022.38361

eMethods.
eTable 1. *ICD-9* and *ICD-10* codes for Jackson Heart Study Coronary Heart Disease Events
eTable 2. Baseline Characteristics of Participants in the Jackson Heart Study by All-Cause Mortality
eTable 3. Baseline Characteristics of Participants in the Jackson Heart Study by Coronary Heart Disease Mortality
eTable 4. Association of Cardiometabolic Multimorbidity With All-Cause Mortality Among Black Adults in the Jackson Heart Study
eTable 5. Association of Cardiometabolic Multimorbidity With Coronary Heart Disease Mortality Among Black Adults in the Jackson Heart Study
eFigure. Exclusion Cascade

This supplemental material has been provided by the authors to give readers additional information about their work.

© 2022 Joseph JJ et al. *JAMA Network Open.*

eMethods.

Methods provided in: Keku E, Rosamond W, Taylor Jr HA, et al. Cardiovascular disease event classification in the Jackson Heart Study: methods and procedures. *Ethn Dis*. 2005;15(suppl 6):S6-62.

<u>Cohort Morbidity and Mortality for CHD Hospitalizations (Morbidity)</u>

To identify CHD events that required hospitalization, myocardial infarction (MI), stroke, or CHF, the annual hospital discharge lists are reviewed for selected International Classification of Diseases, 9th and 10th Revision (ICD9, ICD10) diagnosis codes. The specific codes for each disease category are listed in Table 2. Cohort events are eligible if the participant has: 1) valid cohort identification; 2) events occurred after the baseline examination (visit 1); and 3) an eligible discharge code listed in Table 2 and/or a CHD key word (Table 3) in the discharge summary. Cases that meet these eligibility criteria are investigated by trained and certified abstractors who review all hospital medical records. The abstractors perform medical record abstraction with direct data entry into a laptop computer equipped with Foxpro and all JHS abstraction forms. Abstracted clinical information include presenting symptoms, presence and location of chest pain, history of MI, angina, other CVD, timing of onset of symptoms, use of medication, diagnostic procedures, and therapeutic procedures. The abstractors also recorded cardiac biomarker levels on the first four days of hospitalization or after an in-hospital event. The cardiac biomarkers include troponin I, troponin T, creatinine phosphokinase (CPK) and its isoenzyme CK-MB, serum lactate de- hydrogenase (LDH) and its subfractions LDH1 and LDH2 along with their ratio of LDH1/LDH2. Copies of up to three electrocardiograms (ECG) from the first day, third day (or the first ECG thereafter), and the last day of hospitalization or in-hospital event are obtained and sent to the University of Minnesota ECG Reading Center for classification according to the Minnesota code.8,11,12 The abstracted information and ECG readings are transmitted to the UNC-CSCC at Chapel Hill, North Carolina approximately every two weeks. Abstracted event data are entered into the UNC-CSCC surveillance database and checked for eligibility based on cohort membership, event timing with respect to the baseline clinic exam, and the presence of a valid CVD diagnosis. All incomplete or ineligible forms are referred back to JHS surveillance team for clarification and correction. Other data related to cohort events include computed tomography (CT) and magnetic resonance imaging (MRI) data from hospitalizations for stroke or CHF related events.

In- and Out-of-Hospital Deaths (Mortality)

Deaths are investigated to ascertain that the cause of death is CHD or unspecified CVD. Death certificates that meet the criteria for the underlying cause of death (ICD-9 Codes for identification of CHD deaths: 250,401, 402, 410-414, 427-429, 440, 518.4, 798, 799; ICD-10 codes for ID of CHD deaths: E10-14, I10-11, I21-25, I46-51, I70, I97, J81, J96, R96, R98-99) are reviewed and data are abstracted by a trained abstractor. Hospital records are reviewed for all in-hospital deaths by a trained abstractor. If the deceased had no vital signs on arrival at the hospital or death occurred outside the hospital, the death is classified as an out-of-hospital death. For out-of-hospital death, family member interviews, physician short questionnaires, and coroner records are utilized. A trained abstractor interviews family members about the medical history of the deceased person, the circumstances surrounding the death, and the use of emergency medical services. Contact with the next-of-kin informant that is listed on death certificate is attempted within nine months of the death. A letter is also sent and is followed by a telephone interview to confirm symptoms and events around the time of death. On the average, three informants are contacted for every death certificate, though the informant who is listed on the death certificate often provides the most complete information. Physicians who are familiar with the deceased or the person who signed the death certificate are asked to complete a short questionnaire. If the deceased was hospitalized within 28 days of death, then hospital records

© 2022 Joseph JJ et al. *JAMA Network Open.*

are abstracted. Annual death indices that are provided by the states are used to locate deaths of JHS cohort members. Information on out-of-hospital deaths, including interviews and death certificates, is transmitted to UNC-CSCC every two weeks.

Classification

For MI classification, a computerized algorithm is applied to the hospital record data on symptoms, cardiac biomarkers, and ECG evaluation to generate each participant's computer diagnosis (Table 4). (*White AD, Folsom AR, Chambless LE, et al. Community surveillance of coronary heart disease in the Atherosclerosis Risk in Communities (ARIC). J Clin Epidemiol. 1996; 2:223–233.*) Most cases that have multiple hospitalizations and those with disagreement between the discharge diagnosis code and the computer-generated diagnosis are referred for review or adjudication by the Morbidity and Mortality Classification Committee (MMCC). One MMCC reviewer reviews linked (multiple) and nonlinked hospitalizations when needed. Only certain hospitalized cases get two

MMCC reviewers; however, all out-of-hospital deaths get reviewed independently by two MMCC reviewers. If the two reviewers disagree in their diagnosis, then a trained adjudicator makes the final diagnosis. Using standardized criteria, all out-of-hospital deaths are reviewed and assigned a fatal CHD diagnosis by two MMCC reviewers. (*White AD, Folsom AR, Chambless LE, et al. Community surveillance of coronary heart disease in the Atherosclerosis Risk in Communities (ARIC). J Clin Epidemiol. 1996; 2:223–233.*) The chairman of MMCC adjudicates any disagreement in assigning a final fatal CHD diagnosis. Deaths are classified into: 1) definite fatal MI (requires hospitalization with definite MI within the previous 28 days); 2) definite fatal CHD; 3) possible fatal CHD; 4) non-CHD death; and 5) unclassifiable. Definite and possible CHD deaths are classified according to time from first symptoms to death. The criteria for classifying CHD deaths are based on combinations of: 1) chest pain; 2) history of CHD, MI, or angina; 3) no evidence of other probable cause of death; and/or 4) ICD- 9 codes for identification of CHD deaths: 250, 401, 402, 410-414, 427- 429, 440, 518.4, 798, 799; ICD-10 codes for identification of CHD deaths: E10-14, I10-11, I21-25, I46-51, I70, I97, J81, J96, R96, R98-99.

eTable 1. *ICD-9* and *ICD-10* codes for Jackson Heart Study Coronary Heart Disease Events

**Event Type**	**ICD-9 codes**	**ICD-10 codes**
CHD Death	250, 401, 402, 410-414, 427-429, 440, 518.4, 798, 799	E10-14, I10-11, I21-25, I46-51, I70, I97, J81, J96, R96, R98-99
Hospitalized MI	402, 410-414, 427, 428, 518.4	

© 2022 Joseph JJ et al. *JAMA Network Open.*

eTable 2. Baseline Characteristics of Participants in the Jackson Heart Study by All-Cause Mortality

Baseline Characteristics[a]	Overall	Alive	Deceased All-Cause Mortality	p-value
No. (%) of participants	5,064 (100)	3,996 (79)	1,068 (21)	
Age, mean (SD), y	55.4 (12.8)	52.5 (11.9)	65.7 (10.5)	<0.001
Men	1864 (37%)	1426 (36%)	438 (41%)	0.0013
Women	3200 (63%)	2570 (64%)	630 (59%)	
Less than High School Education	1018 (20%)	603 (15%)	415 (39%)	<0.001
Occupation, Working Full Time	1812 (36%)	1552 (39%)	260 (24%)	<0.001
Current Smoking	665(13%)	483 (12%)	182(17%)	<0.001
AHA Poor Physical Activity[b]	2482 (49%)	1815 (45%)	667 (62%)	<0.001
Alcohol Consumption	2327 (46%)	1958 (49%)	369(35%)	<0.001
Body mass index, mean (SD), kg/m^2	31.8 (7.2)	31.8 (7.2)	31.5 (7.5)	0.1187
Waist Circumference, mean (SD), cm	100.8 (16.1)	100.1 (16.1)	103.1 (16)	<0.001
Systolic Blood Pressure, mean (SD), mmHg	127.5 (16.8)	125.5 (15.5)	135.1 (19.2)	<0.001
Diastolic Blood Pressure, mean (SD), mmHg	75.8 (8.8)	76.1 (8.5)	74.7 (9.5)	<0.001
Hypertension[c]	2864(57%)	2024 (51%)	840 (79%)	<0.001
Low-density lipoprotein, mean (SD), mg/dL, n=4,495	126.6 (36.5)	127 (36.1)	125 (38.2)	0.166
Estimated Glomerular Filtration Rate (CKD-EPI), mean (SD), ml/min per 1.73 m^2	85.9 (18.5)	88.3 (16.4)	76.9 (22.8)	<0.001
Aldosterone, median (IQR), ng/dL	4.4 (2.6, 7.2)	4.3 (2.6, 6.9)	4.6 (2.6, 8.4)	<0.001
Fasting Plasma Glucose, median (IQR), mg/dL, n=4,543	91 (85, 99)	91 (85, 100)	96 (88, 111)	<0.001
Hemoglobin A1C, median (IQR), %, n=4,801	5.7 (5.3, 6.2)	5.6 (5.3, 6)	6.0 (5.5, 6.8)	<0.001
Angiotensin converting enzyme inhibitors	968 (19%)	646 (16%)	322 (30%)	<0.001
Angiotensin Receptor Blockers	413 (8%)	304 (8%)	109 (10%)	0.006
Mineralocorticoid Receptor Antagonists	64 (1%)	38(1%)	26(2%)	<0.001
Statins	574 (11%)	377 (9%)	197 (18%)	<0.001
Diabetes[d]	1108 (22%)	689 (17%)	419 (39%)	<0.001
History of Coronary Heart Disease	374 (7%)	193 (5%)	181(17%)	<0.001
History of Stroke	221 (4%)	108 (3%)	113(11%)	<0.001

© 2022 Joseph JJ et al. *JAMA Network Open.*

Legend eTable 2:

SI conversion factor: To convert low-density lipoprotein cholesterol from milligrams per deciliter to millimoles per liter, multiply by 0.0259; to convert glucose from milligrams per deciliter to millimoles per liter, multiply by 0.0555; to convert HbA1c to the proportion of total Hb, multiply by 0.01; and to convert aldosterone from nanograms per deciliter to picomoles per liter multiply by 27.74

[a] Unless noted otherwise, data are presented as No. (%) of patients. Percentages have been rounded and therefore may not total 100. P-values calculated using chi-square (categorical variables), two sample t-test (parametric continuous variables) and Wilcoxon two sample nonparametric test (non-parametric continuous variables)

[b] AHA = American Heart Association, poor physical activity was defined by AHA “2020” guidelines. Physical Activity was considered poor if participant achieved 0 minutes/week moderate intensity or vigorous intensity physical activity.

[c] Hypertension was defined as systolic blood pressure ≥ 140 mm Hg, diastolic blood pressure ≥ 90 mmHg, or use of antihypertensive therapy.

[d] Diabetes was defined based on 2010 American Diabetes Association guidelines (HbA1c ≥ 6.5%, fasting blood glucose ≥ 126 mg/dL, taking diabetes medications or with a self-reported physician diagnosis

© 2022 Joseph JJ et al. *JAMA Network Open.*

eTable 3. Baseline Characteristics of Participants in the Jackson Heart Study by Coronary Heart Disease Mortality

Baseline Characteristics[a]	Overall	Alive	Deceased CHD Mortality	p-value
No. (%) of participants	5,064 (100)	4,953 (98)	111 (2)	
Age, mean (SD), y	55.4 (12.8)	55 (12.8)	67.4 (9.1)	<0.001
Men	1864 (37%)	1811 (37%)	53 (48%)	0.0157
Women	3200 (63%)	3142 (63%)	58 (52%)	
Less than High School Education	1018 (20%)	972 (20%)	46 (41%)	<0.001
Occupation, Working Full Time	1812 (36%)	1783 (36%)	29 (26%)	0.0319
Current Smoking	665(13%)	647 (13%)	18 (16%)	0.3306
AHA Poor Physical Activity[b]	2482 (49%)	2412 (49%)	70 (63%)	0.0089
Alcohol Consumption	2327 (46%)	2301 (46%)	26 (23%)	<0.001
Body mass index, mean (SD), kg/m^2	31.8 (7.2)	31.8 (7.2)	32 (7.1)	0.6797
Waist Circumference, mean (SD), cm	100.8 (16.1)	100.6 (16.1)	105.9 (16.1)	0.0007
Systolic Blood Pressure, mean (SD), mmHg	127.5 (16.8)	127.4 (16.8)	134.1 (18.1)	<0.001
Diastolic Blood Pressure, mean (SD), mmHg	75.8 (8.8)	75.8 (8.8)	74 (9.7)	0.0332
Hypertension[c]	2864(57%)	2764 (56%)	100 (90%)	<0.001
Low-density lipoprotein, mean (SD), mg/dL, n=4,495	126.6 (36.5)	126.6 (36.4)	124.4 (43.3)	0.6328
Estimated Glomerular Filtration Rate (CKD-EPI), mean (SD), ml/min per 1.73 m^2	85.9 (18.5)	86.2 (18.1)	69.7 (26.3)	<0.001
Aldosterone, median (IQR), ng/dL	4.4 (2.6, 7.2)	4.3 (2.6, 7.2)	5.4 (2.7, 9.4)	0.0137
Fasting Plasma Glucose, median (IQR), mg/dL, n=4,543	91 (85, 99)	91 (85, 100)	99 (89, 21)	<0.001
Hemoglobin A1C, median (IQR), %, n=4,801	5.7 (5.3, 6.2)	5.7 (5.3, 6.1)	6.4 (5.7, 7.5)	<0.001
Angiotensin converting enzyme inhibitors	968 (19%)	924 (19%)	44 (40%)	<0.001
Angiotensin Receptor Blockers	413 (8%)	401 (8%)	12 (11%)	0.3014
Mineralocorticoid Receptor Antagonists	64 (1%)	61 (1%)	3 (3%)	0.1700
Statins	574 (11%)	541 (11%)	33 (30%)	<0.001
Diabetes[d]	1108 (22%)	1044 (21%)	64 (58%)	<0.001
History of Coronary Heart Disease	374 (7%)	329 (7%)	45 (41%)	<0.001
History of Stroke	221 (4%)	202 (4%)	19 (17%)	<0.001

© 2022 Joseph JJ et al. *JAMA Network Open.*

Legend eTable 3:

SI conversion factor: To convert low-density lipoprotein cholesterol from milligrams per deciliter to millimoles per liter, multiply by 0.0259; to convert glucose from milligrams per deciliter to millimoles per liter, multiply by 0.0555; to convert HbA1c to the proportion of total Hb, multiply by 0.01; and to convert aldosterone from nanograms per deciliter to picomoles per liter multiply by 27.74

[a] Unless noted otherwise, data are presented as No. (%) of patients. Percentages have been rounded and therefore may not total 100. P-values calculated using chi-square (categorical variables), two sample t-test (parametric continuous variables) and Wilcoxon two sample nonparametric test (non-parametric continuous variables)

[b] AHA = American Heart Association, poor physical activity was defined by AHA "2020" guidelines. Physical Activity was considered poor if participant achieved 0 minutes/week moderate intensity or vigorous intensity physical activity.

[c] Hypertension was defined as systolic blood pressure ≥ 140 mm Hg, diastolic blood pressure ≥ 90 mmHg, or use of antihypertensive therapy.

[d] Diabetes was defined based on 2010 American Diabetes Association guidelines (HbA1c ≥ 6.5%, fasting blood glucose ≥ 126 mg/dL, taking diabetes medications or with a self-reported physician diagnosis

© 2022 Joseph JJ et al. *JAMA Network Open.*

eTable 4. Association of Cardiometabolic Multimorbidity With All-Cause Mortality Among Black Adults in the Jackson Heart Study

Participants	Full Cohort	No Diabetes, Stroke or Coronary Heart Disease	Diabetes	Stroke	Coronary Heart Disease	Diabetes and Stroke	Coronary Heart Disease and Stroke	Diabetes and Coronary Heart Disease	Diabetes, Stroke, Coronary Heart Disease
Mortality Cases/Total Number	1068/5064	515/3629	297/897	44/104	73/192	31/60	17/31	70/125	21/26
Crude incidence rates per 1,000 person-years (95% CI)	14.79 (13.93, 15.7)	9.65 (8.85, 10.52)	24.40 (21.78, 27.34)	32.95 (24.52, 44.28)	29.07 (23.11, 36.56)	40.18 (28.25, 57.13)	48.02 (29.85, 77.25)	48.62 (38.46, 61.45)	84.06 (54.80, 128.92)
Hazard Ratios (HR)*	Model 0 HR (95% CI, p-value)	ref	1.48 (1.20, 1.81), p<0.001	1.72 (1.23, 2.40), p=0.002	1.61 (1.23, 2.10), p<0.001	1.72 (1.09, 2.71), p=0.019	2.18 (1.32, 3.61), p=0.002	2.27 (1.64, 3.13), p<0.001	3.56 (1.90, 6.68), p<0.001
	Model 1 HR (95% CI, p-value)	ref	1.46 (1.18, 1.80), p<0.001	1.69 (1.21, 2.37), p=0.002	1.58 (1.21, 2.06), p<0.001	1.65 (1.05, 2.61), p=0.031	2.17 (1.30, 3.61), p= 0.003	2.20 (1.58, 3.06), p<0.001	3.31 (1.75, 6.25), p<0.001
	Model 2 HR (95% CI, p-value)	ref	1.41 (1.14, 1.75), p=0.002	1.79 (1.27, 2.54), p=0.001	1.52 (1.14, 2.03), p=0.004	1.53 (0.95, 2.48), p=0.081	2.0 (1.18, 3.38), p= 0.01	2.16(1.53, 3.05), p<0.001	3.08 (1.57, 6.04), p=0.001

Legend eTable 4:

Model 0 – age, sex, education, occupation, smoking, physical activity, alcohol intake and waist circumference, systolic blood pressure, estimated Glomerular Filtration Rate, low-density lipoprotein, hemoglobin A1c + Aldosterone

Model 1 – Model 0 + ACE-Inhibitors, Angiotensin Receptor Blockers, Mineralocorticoid Receptor Antagonists, and Statins

(N=4562 due to missing data on low-density lipoprotein [n=4695] and hemoglobin A1c [n=4968])

Model 2 - Model 1 + Aspirin (n=3688)

© 2022 Joseph JJ et al. *JAMA Network Open.*

eTable 5. Association of Cardiometabolic Multimorbidity With Coronary Heart Disease Mortality Among Black Adults in the Jackson Heart Study

Participants		No Diabetes, Stroke or Coronary Heart Disease	Diabetes	Stroke	Coronary Heart Disease	Diabetes and Stroke	Coronary Heart Disease and Stroke	Diabetes and Coronary Heart Disease	Diabetes, Stroke, Coronary Heart Disease
	Full Cohort								
Mortality Cases/Total Number	111/5064	30/3629	29/897	2/104	9/192	5/60	6/31	24/125	6/26
Crude incidence rates per 1,000 person-years (95% CI)	1.54 (1.28, 1.85)	0.56 (0.39, 0.80)	2.38 (1.66, 3.43)	1.50 (0.37, 5.99)	3.58 (1.86, 6.89)	6.48 (2.70, 15.57)	16.95 (7.61, 37.73)	16.67 (11.17, 24.87)	24.02 (10.79, 53.46)
Hazard Ratios (HR)*	Model 0 HR (95% CI, p-value)	ref	1.89 (0.94, 3.83) p=0.076	1.22 (0.28, 5.22) p=0.790	3.53 (1.57, 7.96) p=0.002	5.63 (1.98, 15.99) p=0.001	10.55 (3.96, 28.13) p<0.001	13.73 (6.67, 28.23) p<0.001	12.14 (3.06, 48.13) p<0.001
	Model 1 HR (95% CI, p-value)	ref	1.84 (0.90, 3.76), p=0.092	1.20 (0.28, 5.15), p=0.810	3.47 (1.53, 7.84), p=0.003	5.32 (1.86, 15.21), p=0.002	9.97 (3.66, 27.18), p<0.001	13.27 (6.26, 28.14), p<0.001	11.22 (2.84, 44.33), p<0.001
	Model 2 HR (95% CI, p-value)	ref	1.78 (0.85, 3.72), p=0.126	1.3 (0.3, 5.64), p=0.728	2.24 (0.84, 6.02), p=0.109	5.13 (1.77, 14.9), p=0.003	6.96 (2.3, 21.05), p<0.001	11.48 (5.11, 25.77), p<0.001	10.83 (2.62, 44.73), p<0.001

Legend eTable 5:

Model 0 – age, sex, education, occupation, smoking, physical activity, alcohol intake and waist circumference, systolic blood pressure, eGFR, LDL, Hemoglobin A1C + Aldosterone

Model 1 – Model 0 + ACE-Inhibitors, Angiotensin Receptor Blockers, Mineralocorticoid Receptor Antagonists, and Statins

(N=4562 due to missing data on low-density lipoprotein [n=4695] and hemoglobin A1c [n=4968])

Model 2- Model 1+Aspirin (n=3688)

© 2022 Joseph JJ et al. *JAMA Network Open.*

eFigure. Exclusion Cascade

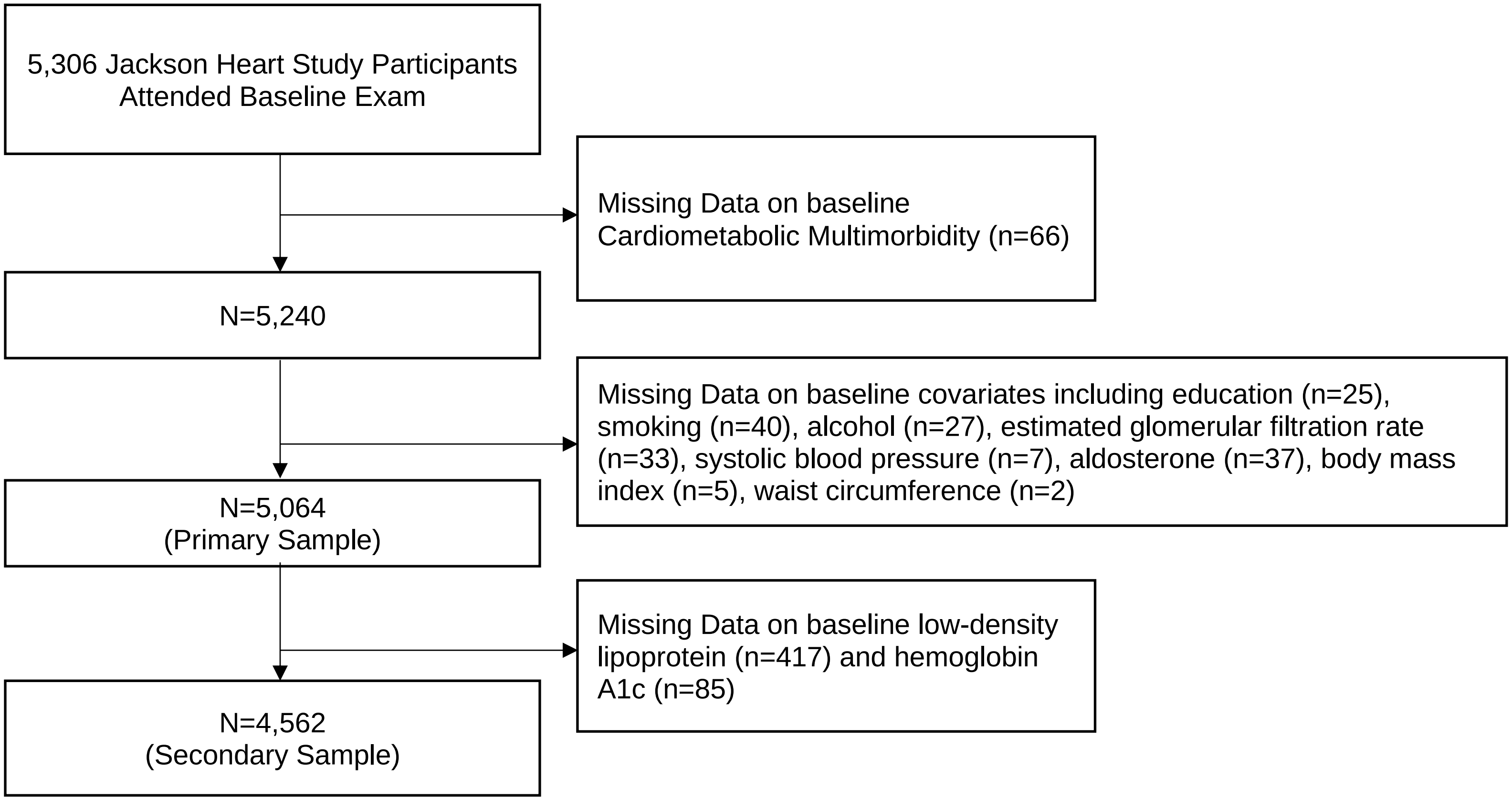

© 2022 Joseph JJ et al. *JAMA Network Open.*
